# Analyses of Ferrous and Ferric State in Dynabi_Tab_ Using Mössbauer Spectroscopy

**DOI:** 10.1155/2017/9321896

**Published:** 2017-04-09

**Authors:** Young Rang Uhm, Jae Cheong Lim, Sang Mu Choi

**Affiliations:** Nuclear Materials Research Division, Korea Atomic Energy Research Institute (KAERI), Daejeon 34057, Republic of Korea

## Abstract

Antianemic medicament ferrous gluconate, ferrous fumarate, and a Dynabi tablet with a basic iron bearing ingredient were studied with the use of Mössbauer spectroscopy. Room temperature spectra of ferrous gluconate gave clear evidence that the two phases of iron were present: ferrous (Fe^2+^) as a major one with a contribution at and above 91 a.u.% and ferric (Fe^3+^) whose contribution was found to be ~9 a.u.%. In the case of ferrous fumarate, a single phase was measured corresponding to ferrous (Fe^2+^) state. A Dynabi tablet consists of ferrous fumarate and ferrous fumarate. The ferric phase in ferrous gluconate is able to be reached about ~3.6 a.u.% in a tablet.

## 1. Introduction

Mössbauer spectroscopy is widely used for studying various molecular including biomolecular systems as well as various materials containing Mössbauer isotopes such as ^57^Fe, ^119^Sn, ^121^Sb, ^127^I, and ^197^Au. The nuclear hyperfine field, quadrupole splitting, and isomer shift provide very precise information about the electronic and magnetic state of the nuclei, chemical bonds, structure of the local environment, and so on [1, 2]. A number of pharmaceutical compounds contain Mössbauer atoms such as Fe, Sn, and Au. Pharmaceuticals containing Fe are mainly used for iron deficiency treatment. Iron is a very important mineral in the organic system in the human body. This element is an integral part of many proteins and enzymes. In addition, iron is essential for the regulation of cell growth and differentiation. Iron deficiency causes anemia and other pathological changes in the body. Absorption of iron from food requires recognition of the chemical form of iron by gut receptors. Both the shape and charge are important in the recognition process. Iron in a dietary supplement should be ferrous (Fe^2+^) to be absorbed in the body because it is soluble. The ferric Fe^3+^ ions are chelated, such as citrate, phytate, and heme [3]. Therefore, the iron valence state is very important information because it may be related to the effect and toxicity of pharmaceutical products.

Various vitamins and dietary supplements contain Fe in the form of ferrous fumarate, ferrous sulfate, and ferrous gluconate. Recently several commercial samples of Elevit (Rottendorf Pharma GmbH, Germany), Vitrum (Unifarm, Inc., USA), Children's Multi Vitamin (Target Corporation Minneapolis, USA), Multi For Her (Nature Made Nutritional Products, USA), and Multitabs (Ferrosan A/S, Denmark) containing ferrous fumarate and Sorbifer Durules (Egis, Hungary), Hemofer® (Glaxo-SmithKline Medicines S.A., Poland), Falvit® (Jelfa, Poland), and Vitaral (Jelfa, Poland) containing ferrous sulfate were studied using Mössbauer spectroscopy [4–6].

In this study, standard samples of ferrous fumarate and gluconate were measured using Mössbauer spectroscopy. The iron valence state and dietary supplements of a Dynabi tablet containing both ferrous fumarate and ferrous gluconate were selected and measured. A tablet of Dynabi is a famous iron supplement (dietary supplement) produced by Korean pharmaceutical company used to treat anemia or other iron deficiencies. It is used to be prescribed to pregnant and parturient women, occasionally in Korea (South).

## 2. Experimental

Mössbauer spectra were measured in a transmission geometry with a moving absorber at a temperature of 295 K and recorded in 1024 channels. For their analysis, spectra with a low iron content and poor signal-to-noise ratio were converted into 512 channels [7]. The spectrometer velocity was calibrated with a high-purity *α*-^57^Fe foil. The studied dietary supplement contains iron compounds per tablet (275 mg in Dynabi), which is produced by Dong-A pharmaceutical company in Republic of Korea. The reference compounds of both ferrous gluconate and ferrous fumarate were commercial products. Both compounds are produced by Spectrum Chemical Manufacturing Corporation in USA. All samples consisting of 100 mg powder were distributed homogeneously on a surface of ~3.2 cm^2^.

The Mössbauer investigation was made on powdered Dynabi samples. The reference materials such as ferrous fumarate and gluconate were measured as powder.

## 3. Results and Discussion

**Figure 1:**
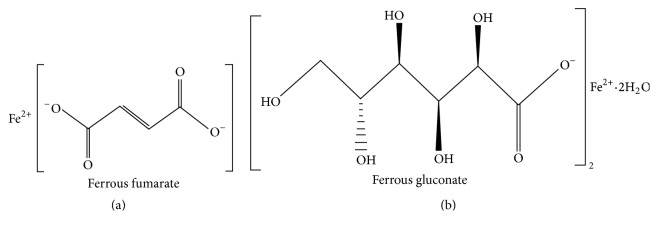
Chemical structure of (a) ferrous fumarate and (b) ferrous gluconate [8].

The molecular formulas of the ferrous fumarate and ferrous gluconate are C_4_H_2_FeO_4_ and C_12_H_22_FeO_14_·2H_2_O, respectively. The chemical structures are represented at Figure 1.

**Figure 2:**
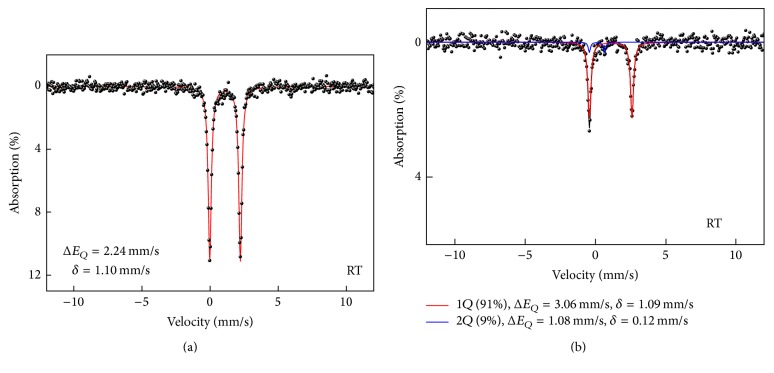
The room temperature Mössbauer spectra of (a) ferrous fumarate and (b) ferrous gluconate.

The room temperature Mössbauer spectra for the samples are fitted a doublet. However, according to a certificate analysis for the commercial ferrous gluconate, the ferric iron is included at about 2 wt.%. Thus, the Mössbauer spectrum for ferrous gluconate is fitted as two phases. The room temperature Mössbauer spectra of ferrous fumarate and gluconate are presented in Figure 2. The fitting parameters of all spectra are listed in Table 1.

The room temperature Mössbauer spectrum for ferrous fumarate is fitted as one doublet with a ferrous state, only as shown in Figure 2(a). The Mössbauer spectrum for ferrous gluconate is fitted as two sets of doublets, as shown in Figure 2(b). The separation of the ferrous and the ferric phases is based on values of two parameters such as isomer shifts (IS) and quadrupole splitting (QS). Their values depend not only on the valence state but also on the spin states of the low spin (LS) and high spin (HS) [9]. In ferrous gluconate, the minor phase is contributed by either the ferrous-LS or ferric-HS. The recoil free fractions (*f*-factor) of the ferrous and the ferric are different. However, this value is fixed at same valent state, though the spin states are different. Both IS and QS for the ferric ion are overlapped with ferrous-LS. However, the identification of the spin state and valence state of the minor phase in ferrous gluconate is unclear, yet [10, 11]. The surface integral of the fitted spectrum is applied to the composition of the sample. The area of the spectrum corresponds to atomic percentage (a.u.%) of the phase in the sample. For the inorganic compounds, the overestimation for the ferric phase was reported to be as high as 15%. A ferric or Fe^3+^ (LS) phase in the ferrous gluconate reaches ~9 a.u.% in the sample. The peaks corresponding to ferrous iron show a slightly weaker Lamb-Mössbauer factor than those of the ferric phase [9]. These doublets show different values of quadrupole splitting (Δ*E*_*Q*_) and isomer shifts (*δ*). Additional components connected with Fe^3+^ in samples may be considered as an impurity or a result of ferrous gluconate oxidation and the formation of ferric gluconate [8].

**Figure 3:**
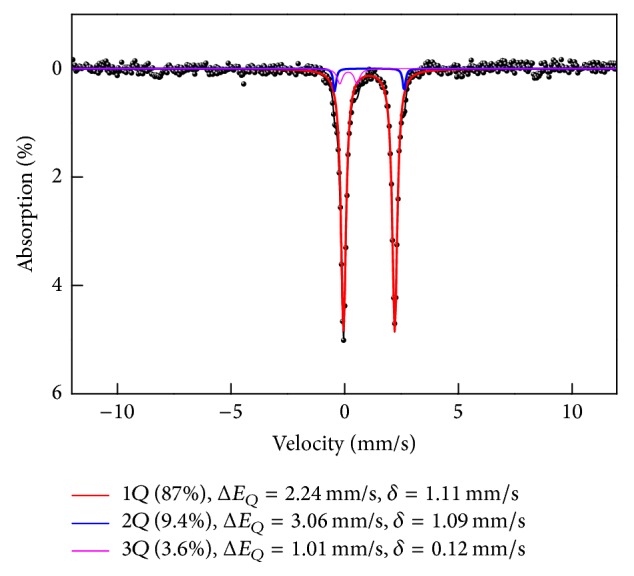
Mössbauer spectroscopy to study an industrial sample Dynabi tablet including ferrous fumarate and ferrous gluconate.

The room temperature Mössbauer spectrum of a dietary supplement containing both ferrous fumarate and ferrous gluconate is presented in Figure 3. This spectrum was fitted by tree doublets. The fitting parameters in the spectrum are listed in Table 2.

The parameters of the two doublets are connected with ferrous gluconate (C_12_H_22_FeO_14_·2H_2_O). The parameters of the spectrum such as quadrupole splitting and isomer shifts are same as those of pure ferrous gluconate. The remaining doublet is related to ferrous fumarate (C_4_H_2_FeO_4_). The quadrupole splitting for the ferrous fumarate shows a lower value than those of the Fe^2+^ compound of ferrous gluconate because of a slightly different symmetry. The doublet with *δ* and Δ*E*_*Q*_ values of 1.11 and 2.25 mm/s is attributed to ferrous fumarate. A tablet of Dynabi consists of ferrous fumarate 175 mg (0.97 mole), ferrous gluconate 100 mg (0.21 mole), folic acid 0.4 mg (0.001 mole), and ascorbic acid 309 mg (1.75 mole). Mössbauer spectrum reveals only an iron connected phase. According to a certificate analysis of the commercial product, the molar ratios for ferrous fumarate and ferrous gluconate are above 82.3 and 17.7 a.u.%, respectively. However, the areas of ferrous fumarate and gluconate are fitted to be 87 and 13%, as shown in Table 2. In addition, the area of the ferric phase reached to be ~3.6%. Though the Fe^2+^/Fe^3+^ ratio obtained from spectra area of the subspectra does not contribute real concentration of the phase, the overestimation is to be as high as 15% [9]. The absorption area is able to be converted to ~27.7 a.u.% in ferrous gluconate. The ferric phase is significantly decreased compared with pure ferrous gluconate of ~9 a.u.%.

We may suppose that the doublet with *δ* and Δ*E*_*Q*_ values of 0.12 and 0.9 mm/s is attributed to a ferric phase in ferrous gluconate, according to a certificate analysis. This results in ferrous gluconate oxidation and the formation of ferric gluconate [10, 11]. The doublet with *δ* and Δ*E*_*Q*_ values of 1.09 and 3.06 mm/s is attributed to ferrous gluconate.

## 4. Conclusion

The results of the applications of Mössbauer spectroscopy to study industrial samples such as ferrous fumarate, ferrous gluconate, and a dietary supplement demonstrate a wide possibility of this technique. ^57^Fe hyperfine parameters of the studied pharmaceuticals indicate the existence of major iron ferrous and ferric (or ferrous-LS) compounds. The studied dietary supplement consists of ferrous fumarate (above 87 a.u.%) and gluconate (~13 a.u.%). The Mössbauer spectrum estimated the presence of a ferric compound, owing to impurity and partially modified ferrous gluconate.

## Figures and Tables

**Table 1:** The parameters of quadrupole splitting (Δ*E*_*Q*_), isomer shifts (*δ*), and absorption area for ferrous gluconate and ferrous fumarate.

		Δ*E*_*Q*_ (mm/s)	*δ* (mm/s)	Area (%)
Gluconate				
Ferrous 1		3.06	1.09	91
Ferric		1.08	0.12	9
Fumarate				
Ferrous 2		2.24	1.11	100

**Table 2:** The parameters of quadrupole splitting (Δ*E*_*Q*_), isomer shifts (*δ*), and absorption area for Dynabi_Tab_.

	Δ*E*_*Q*_ (mm/s)	*δ* (mm/s)	Area (%)
Dynabi_Tab_			
Ferrous 1	3.06	1.09	9.4
Ferrous 2	2.24	1.11	87
Ferric	1.01	0.12	3.6
